# ED50 of ciprofol combined with sufentanil for fiberoptic bronchoscopy of different patient populations with pulmonary tuberculosis

**DOI:** 10.1186/s12871-024-02583-w

**Published:** 2024-06-04

**Authors:** Min Pan, Weidong Liu, Zhixin Zhang, Tong Li, Weibin Xie

**Affiliations:** 1 Department of Pharmacy, The Third People’s Hospital of Changzhou, Changzhou, Jiangsu 213001 China; 2 Department of Anesthesiology, The Third People’s Hospital of Changzhou, Changzhou, Jiangsu 213001 China; 3 Department of Tuberculosis, The Third People’s Hospital of Changzhou, Changzhou, Jiangsu 213001 China; 4 Department of Administrative Office, The Third People’s Hospital of Changzhou, Changzhou, China; 5 Changzhou Medical Center, Nanjing Medical University, Changzhou, China

**Keywords:** Ciprofol, ED50, Fiberoptic bronchoscopy, Pulmonary tuberculosis, Dixon’s up-and-down method

## Abstract

**Background:**

Ciprofol is a promising sedative. This study aims to explore the median effective dose (ED50) of ciprofol in inhibiting responses to fiberoptic bronchoscopy in patients with pulmonary tuberculosis (PTB) of different genders and ages when combined with 0.15 μg/kg sufentanil, and to evaluate its efficacy and safety, providing a reference for the rational use of ciprofol in clinical practice.

**Methods:**

PTB patients who underwent bronchoscopy examination and treatment at The Third People’s Hospital of Changzhou between May 2023 and June 2023 were selected and divided into four groups using a stratified random method. All patients received intravenous injection of 0.15 μg/kg sufentanil followed by injection of the test dose of ciprofol according to Dixon’s up-and-down method. The initial dose of ciprofol in all four groups was 0.4 mg/kg, with an adjacent ratio of 1:1.1. The next patient received a 10% increase in the dose of ciprofol if the previous patient in the same group experienced positive reactions such as choking cough, frowning, and body movements during the endoscopy. Otherwise, it was judged as a negative reaction, and the next patient received a 10% decrease in the dose of ciprofol. The transition from a positive reaction to a negative reaction was defined as a turning point, and the study of the group was terminated when seven turning points occurred. Hemodynamic parameters, oxygen saturation and adverse reactions were recorded at different time points in all groups. The Probit regression analysis method was used to calculate the ED50 of ciprofol in the four groups and compare between the groups.

**Results:**

The ED50 of ciprofol combined with 0.15 μg/kg sufentanil for bronchoscopy in the four groups were 0.465 mg/kg, 0.433 mg/kg, 0.420 mg/kg and 0.396 mg/kg, respectively.

**Conclusion:**

The ED50 of ciprofol used for fiberoptic bronchoscopy varied among PTB patients of different genders and ages.

**Trial registration:**

The Chinese Clinical Trial Registry, ChiCTR2300071508, Registered on 17 May 2023.

## Introduction

Fiberoptic bronchoscopy has gradually become an important diagnostic and therapeutic procedure in the treatment of pulmonary tuberculosis (PTB). Clinical studies both at home and abroad have confirmed that the positive rate of pulmonary tuberculosis diagnosed by etiology and pathology of respiratory tract specimens obtained through bronchoscopy is higher than that of sputum specimens [1]. The operation time for fiberoptic bronchoscopy is relatively short (several minutes to tens of minutes), but it can cause strong airway stimulation, leading to coughing, fear, and other discomfort in patients. Currently, most guidelines or expert consensus both at home and abroad suggest administering moderate sedation and anesthesia during tracheoscopy to improve patient tolerance and satisfaction [2, 3]. The consensus among experts from the Chinese Society of Anesthesiology (CSA) clearly requires routine intraoperative monitoring of four indicators: electrocardiogram (ECG), respiratory rate (RR), non-invasive blood pressure (BP), and oxygen saturation (SpO2).

Patients with PTB often experience weight loss, cough, chest pain and low immunity [4]. Rifampicin is the main drug used to treat tuberculosis. However, rifampicin is a liver enzyme inducer that can reduce the blood concentration and effectiveness of certain drugs, thereby affecting the therapeutic effect of the disease [5]. Clinically, we have observed that the dosage of ciprofol may need to be increased in patients with tuberculosis due to rifampicin use. However, further verification and exploration are needed to determine the specific pathway and mechanism of action. Therefore, sedation and analgesia have always been challenging during bronchoscopy for PTB patients [6, 7].

Ciprofol, a 2,6-phenol derivative, is a promising sedative with the advantages of rapid onset, high therapeutic index, low incidence of respiratory and cardiac function inhibition, reduced injection pain, and suitability for all kinds of endoscopic diagnosis and treatment [8–10]. After entering the blood, ciprofol is distributed widely in tissues with high fat content due to its fat-soluble nature. The pharmacokinetics of ciprofol exhibit characteristics such as high clearance and short half-life. Approximately 95% of ciprofol in the blood binds to plasma protein while 5% remains unbound. The liver serves as the primary site for ciprofol metabolism, resulting in various metabolites detectable in plasma, urine, and feces; among these metabolites glucuronide conjugate is the most significant [8, 11]. Previous studies on the efficacy and safety of ciprofol have demonstrated that a dosage range of 0.5–0.6 mg/kg alone can fulfill sedation/anesthesia requirements for gastrointestinal endoscopy [12]. When used in combination with opioid analgesics, it is recommended to administer an initial dose of 0.4 mg/kg followed by additional doses ranging from 0.15 to 0.2 mg/kg each time. In patients undergoing fiberoptic bronchoscopy procedures, ciprofol at a dosage range between 0.3–0.4 mg/kg exhibits comparable sedative/anesthetic effects compared to propofol at dosages ranging from 1.2–2 mg/kg; however, ciprofol at a dosage level of 0 0.4 mg / kg has the longest induction time [13, 14]. Ciprofol has been used in clinical practice for a short period of time, and there are limited studies available for minors, patients with special diseases, as well as acute and critical patients. The median effective dose (ED50) of ciprofol has not been reported for specific populations such as PTB patients. A previous pharmacokinetic study conducted on the healthy Chinese population revealed that the ED50 of ciprofol differed between non-elderly and elderly individuals [15]. Therefore, in this study, the sequential test method was used to further study the ED50 of ciprofol in bronchoscopy of PTB patients of different genders and ages, in order to provide reliable reference and basis for clinical use of ciprofol.

## Methods

### Patients and clinical protocol

This was a prospective study with a sequential design, approved by the Ethics Committee of The Third People’s Hospital of Changzhou (Approval No. 02A-A20230004). This study was registered with the Chinese Clinical Trial Registry (ChiCTR2300071508) in 17/05/2023 and informed consent form was signed by all the patients. This study adhered to the CONSORT 2010 statement.

PTB patients who were classified as Grade II by the American Society of Anesthesiologist (ASA), with a Body Mass Index (BMI) 18–28 kg/m^2^, regardless of age and gender and underwent bronchoscopy examination or treatment at The Third People’s Hospital of Changzhou between May 2023 and June 2023 were included. The non-elderly group was 18–64 years old, while the elderly group was ≥ 65 years old in this study [15, 16]. The exclusion criteria were as follows: patients with recent major hemoptysis, severe stenosis of the main airway, myasthenia gravis, severe cardiovascular disease, long-term history of taking psychotropic drugs, allergies to anesthetic drugs and their components, and estimated surgical duration exceeding 30 min.

### Study design

All patients did not receive any preoperative medication. After entering the room, the patient was supine for five minutes after which the upper limb vein channel was opened, and ECG, BP and SpO_2_ were routinely monitored. The enrolled patients in this study were divided into four groups according to a stratified random method: Group N1 (non-elderly male patients), Group N2 (non-elderly female patients), Group N3 (elderly male patients), and Group N4 (elderly female patients). After intravenous injection of 0.15 μg/kg sufentanil, all patients in this study were sedated with the test dose of ciprofol. Based on pre-experiment results and package insert, the initial dose of ciprofol in all four groups was 0.4 mg/kg, and the administration time was ≥ 30 s. The examination was performed after the patient’s consciousness and eyelash reflex had disappeared. The experiment was conducted using Dixon’s up-and-down method, with a dose gradient ratio of 1:1.1 for two adjacent patients in each group [17–19]. If the previous patient showed a positive reaction during bronchoscopy (such as choking cough, frowning, body movements, etc.), immediate remedial measures were undertaken (an additional 0.15 mg/kg of ciprofol was added to enhance sedation), and the next patient in the same group received a 10% increase in ciprofol. In contrast, in case of a negative reaction, the next patient in the same group received a 10% decrease in ciprofol until seven turning points were reached, then the trial in this group was terminated.

All fiberoptic bronchoscopy operations in this study were performed by the same chief physician of the PTB department, and the anesthesia procedures were performed by the same anesthesiologist. When the patient’s systolic blood pressure (SBP) was ≤ 90 mmHg during anesthesia, intravenous injection of 5 mg ephedrine was given; when heart rate (HR) ≤ 60 times/min, intravenous atropine 0.5 mg was given. When SpO_2_ ≤ 90%, the inhalation oxygen flow rate was increased or the jaw was raised. Sufentanil and ciprofol used in this study were produced by Yichang Humanwell Pharmaceutical (batch number: 21A04051) and Liaoning Haisike Pharmaceutical (batch number: 20220810).

### Observation indexes

Positive/negative reactions, intraoperative vital signs and postoperative adverse reactions of all patients were recorded by another anesthesiologist who was unaware of the dosage of ciprofol.

The primary outcome of this study was the ED50 and the 95% effective dose (ED95) of ciprofol in the four groups. Secondary outcomes included SBP, diastolic blood pressure (DBP), HR, SpO_2_ before anesthesia induction (T1), when eyelash reflex disappeared (T2), when fiberoptic bronchoscope was inserted (T3), and after awakening (T4), and intraoperative hypotension (SBP ≤ 90 mmHg), bradycardia (HR ≤ 60 beats/min), respiratory depression (SpO_2_ ≤ 90%), and postoperative adverse reactions such as injection pain, nausea, vomiting, headache and dizziness.

### Statistical analysis

Based on the results of our preliminary study, we assumed that the success rates of sedation from ciprofol would be 90%. Considering a non-inferiority margin of 20%, a power of 80%, and a one-sided alpha level of 2.5%, the required sample size for each group was determined to be 40. However, due to the non-independence (where the dose administered to the next patient depended on the response of the previous patient) and unknown dose distribution, we recruited 45 subjects for each group in this study [14].

Statistical analysis was conducted using SPSS 23.0 software. Data of the patients was collected. The continuous variables with normal distribution were expressed as mean ± standard deviation and compared by ANOVA. The measurement data with non-normal distribution were represented by the median (interquartile interval, IQR) and analyzed by Mann–Whitney U test. Probit regression analysis was used to calculate the ED50 and ED95 of ciprofol and their corresponding 95% confidence intervals (CI). A *p value* < 0.05 was considered statistically significant. The sample size of this study was based on α = 0.05 for the two-sided chi-square test to analyze trends in proportions and a logistic model of β = 0.1 to detect the success rates.

## Results

**Fig. 1:**
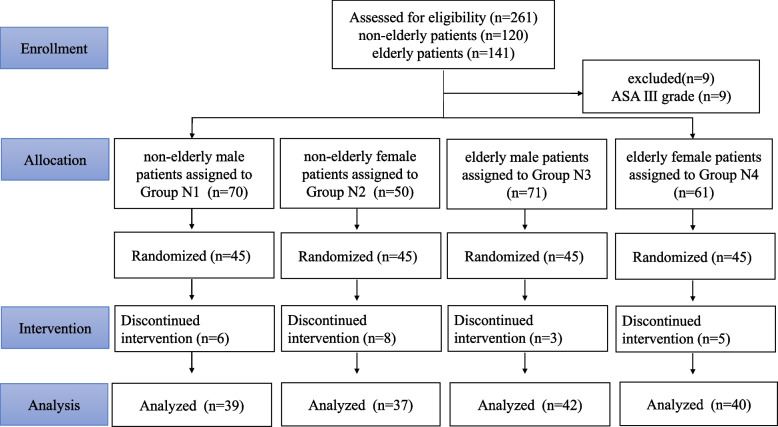
Flowchart of patient enrollment, allocation, intervention and analysis

From May 2023 to June 2023, 261 PTB patients who underwent elective bronchoscopy or treatment at The Third People’s Hospital of Changzhou were evaluated, of which 9 patients with ASA grade III were excluded since they did not meet the inclusion criteria (Fig. 1). Finally, 180 patients were included and stratified into four groups, with 45 patients in each group. The study was terminated after 39 patients in Group N1, 37 in Group N2, 42 in Group N3 and 40 in Group N4 completed seven turning points (Table 1).

**Table 1:** The general characteristics of patients in the four groups

Values	Group N1	Group N2	Group N3	Group N4	*P*-value
Number of Interventions	39	37	42	40	0.447
Effective/ Ineffective	19/20	18/19	21/21	20/20	0.999
Age (years)	52.00 (46.50, 56.00)	52.50 (44.25, 56.00)	70.00 (67.00, 75.00)	70.00 (68.00, 76.50)	0.000
BMI (kg/m^2^)	21.46 (19.14, 23.16)	19.99 (18.46, 21.35)	20.86 (19.44, 22.14)	20.28 (18.64, 22.15)	0.725

Values were expressed as the median (IQR) or the number of patients

*BMI* body mass index

The ED50 and ED95 of ciprofol combined with sufentanil for bronchoscopy in non-elderly male PTB patients were 0.465 mg/kg (95% CI: 0.414–0.518 mg/kg) and 0.598 mg/kg (95% CI: 0.535–1.003 mg/kg), respectively.

The ED50 and ED95 of ciprofol combined with sufentanil for bronchoscopy in non-elderly female PTB patients were 0.433 mg/kg (95% CI: 0.405–0.466 mg/kg) and 0.517 mg/kg (95% CI: 0.478–0.694 mg/kg), respectively.

The ED50 and ED95 of ciprofol combined with sufentanil for bronchoscopy in elderly male PTB patients were 0.420 mg/kg (95% CI: 0.378–0.462 mg/kg) and 0.538 mg/kg (95% CI: 0.483–0.846 mg/kg), respectively.

The ED50 of ciprofol combined with sufentanil for bronchoscopy in elderly female PTB patients was 0.396 mg/kg (95% CI: 0.366–0.427 mg/kg), and the ED95 was 0.482 mg/kg (95% CI: 0.443–0.667 mg/kg).

**Fig. 2:**
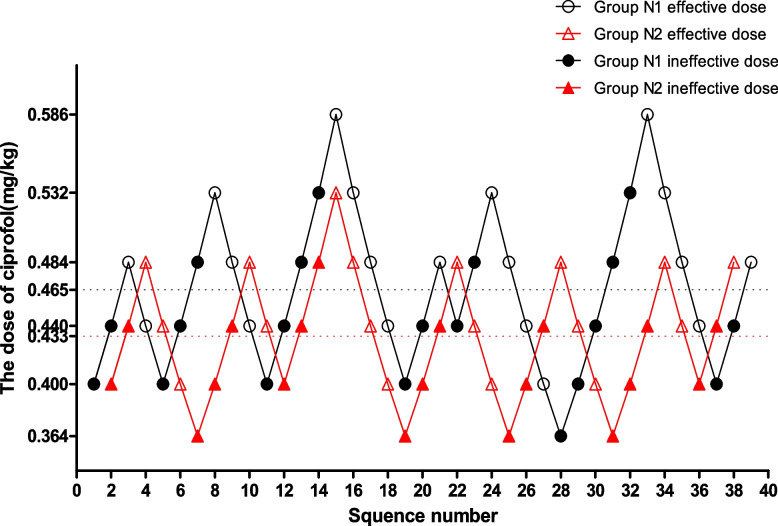
Sequential dose adjustment of ciprofol when combined with 0.15 μg/kg sufentanil by the Dixon method in the N1 and N2 groups. The open circle and the open triangle represent an effective dose; the filled circle and the filled triangle indicate an ineffective dose. The ED50 of ciprofol in the N1 and N2 groups were 0.465 mg/kg and 0.433 mg/kg, respectively

**Fig. 3:**
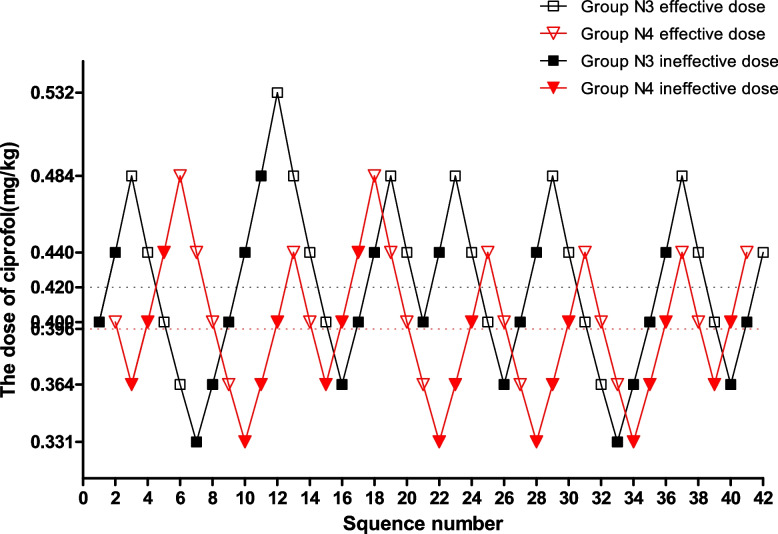
Sequential dose adjustment of ciprofol when combined with 0.15 μg/kg sufentanil by the Dixon method in the N3 and N4 groups. The open square and the open inverted triangle represent an effective dose; the filled square and the filled inverted triangle indicate an ineffective dose. The ED50 of ciprofol in the N3 and N4 groups were 0.420 mg/kg and 0.396 mg/kg, respectively

The sequential dose test of ciprofol combined with sufentanil for bronchoscopy in the non-elderly and elderly PTB patients is shown in Figs. 2 and 3, respectively.

The ED50 of ciprofol in Group N1 was significantly higher than in Group N2 (0.465 mg/kg vs. 0.433 mg/kg, *p* = 0.007) and Group N3 (0.465 mg/kg vs. 0.420 mg/kg, *p* = 0.000). The ED50 of ciprofol in Group N4 was significantly lower than in Group N2 (0.396 mg/kg vs. 0.433 mg/kg, *p* = 0.001) and Group N3 (0.396 mg/kg vs. 0.420 mg/kg, *p* = 0.020).

The incidence of dizziness and headache in Group N1 was slightly higher than in Groups N2, N3, and N4, and the incidence of injection pain was slightly lower compared to Groups N2, N3, and N4, however, none of them were statistically significant (*p* > 0.05). There was no significant difference in the incidence of hypotension, bradycardia, respiratory depression, nausea and vomiting among the four groups (*p* > 0.05) (Table 2).

**Table 2:** Comparison of adverse events among the four groups

Values	Group N1	Group N2	Group N3	Group N4	*P*-value
Hypotension	0/39 (0%)	0/37 (0%)	0/42 (0%)	1/40 (2.50%)	0.402
Bradycardia	0/39 (0%)	1/37 (2.70%)	0/42 (0%)	0/40 (0%)	0.354
Respiratory depression	0/39 (0%)	0/37 (0%)	0/42 (0%)	0/40 (0%)	
Injection pain	4/39 (10.26%)	5/37 (13.51%)	6/42 (14.29%)	5/40 (12.50%)	0.956
Nausea and vomiting	1/39 (2.56%)	0/37 (0%)	0/42 (0%)	0/40 (0%)	0.387
Dizziness and headache	9/39 (23.08%)	7/37 (18.92%)	8/42 (19.05%)	7/40 (17.50%)	0.936

Values are expressed as the number of patients and percentage

**Fig. 4:**
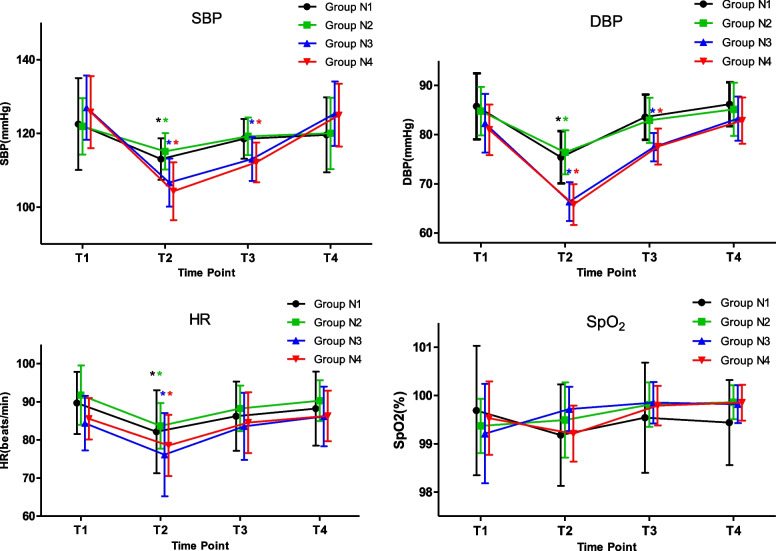
Comparison of hemodynamic parameters and oxygen saturation among the four groups at different time points. SBP, systolic blood pressure; DBP, diastolic blood pressure; HR, heart rate; SpO2, oxygen saturation. T1: before anesthesia induction; T2: when eyelash reflex disappeared; T3: when fiberoptic bronchoscopy was inserted; T4: after awakening. Compared with T1, *p < 0.05

The changes in hemodynamics and oxygen saturation of the four groups at different time points are shown in Fig. 4. The four groups showed significant differences in SBP, DBP, and HR between T1 and T2 (*p* < 0.05). The SBP and DBP of the elderly groups in T3 were significantly lower than in T1 (*p* < 0.05). There was no significant difference in oxygen saturation at different time points in each group (*p* < 0.05).

## Discussion

Patients undergoing fiberoptic bronchoscopy require sedatives to enhance comfort, alleviate anxiety, and reduce surgical complications [14]. Ciprofol is a short-acting intravenous sedative based on the structural modification of propofol. Its main mechanism is to activate the postsynaptic GABA-A-Cl-channel complex, which causes hyperpolarization of nerve cell membrane through chloride ion influx, thus causing central nervous system inhibition [11, 20]. The highly selective binding ability of ciprofol to its receptor enables it to achieve the same sedative and anesthetic effects as propofol at a lower dose [21–24].

Currently, the efficacy and safety of ciprofol have been recognized. Ciprofol exhibits less inhibition on the respiratory system compared to propofol and is more stable in hemodynamics. Due to its poor water solubility, ciprofol is formulated into an oil-in-water emulsion with a lower concentration, resulting in a significantly reduced risk of injection pain compared to propofol [25–27]. However, given its short time in the market, there is no reference dose for the use of ciprofol in special populations with low immunity such as PTB patients. On the other hand, it has not been reported whether the use of rifampicin (a liver enzyme inducer) affects the median effective dose of cipofol. Therefore, this study prospectively included PTB patients and calculated the ED50 of ciprofol combined with 0.15 μg/kg sufentanil for fiberoptic bronchoscopy examination of non-elderly men, non-elderly women, elderly men, and elderly women by sequential test method, in order to provide a reliable basis for the effectiveness of ciprofol. The ED50 of ciprofol in non-elderly male and female patients (0.465 mg/kg and 0.433 mg/kg) in this study was slightly higher than the ED50 of ciprofol in non-elderly patients published by Luo et al. (0.4 mg/kg). This difference might be attributed to the fact that the patients included in their study were administered 2% lidocaine inhalation (10 mL) within one hour before receiving ciprofol, or it could be related to the daily intake of rifampicin among tuberculosis patients in this study [13].

This study also found a significant difference in the ED50 of ciprofol for PTB patients of different genders and ages, and the ED50 of ciprofol in elderly patients were significantly lower than the non-elderly patients, and the ED50 of ciprofol in the female patients were significantly lower than in the male patients, suggesting that there may be age and gender differences in the pharmacokinetics and pharmacodynamics of ciprofol, which was consistent with Li et al.’s conclusion that the use of 0.3 mg/kg of ciprofol in elderly people was equally effective as the use of 0.4 mg/kg in non-elderly people [15]. It was also consistent with the conclusion reported by Duan et al. that the recommended use of low-dose (0.3 mg/kg) ciprofol for anesthesia induction in elderly patients had better safety and effectiveness [16], which may be related to weakened liver and kidney functions and cardiovascular system decline in the elderly [11, 28]. Elderly patients have lower myocardial contractility, coronary blood flow, and ventricular compliance, which decreases the tolerance to excessive volume loading [29]. Moreover, elderly patients have lower stress adaptation ability to surgical anesthesia and higher responsiveness to anesthetic drugs [15]. Gender differences have varying degrees of impact on the absorption, distribution, and metabolism of drugs, which may be related to estrogen and metabolic enzymes in women [30, 31], but the specific mechanisms need further study.

The incidence of hypotension, bradycardia, and respiratory depression of 158 patients in the four groups was very low, which may be related to the reduction of oxidative damage, inflammatory response, and myocardial cell apoptosis caused by ciprofol [32, 33]. Moreover, hemodynamic fluctuations were more pronounced in the elderly group than in the non-elderly group.

In this study, the combination of ciprofol and sufentanil was selected. The peak time of sufentanil and ciprofol blood concentration was 3–5 min and 2–3 min, respectively [34, 35]. Therefore, first sufentanil was slowly pushed for one minute, after which ciprofol was slowly pushed for one minute. Then we waited for about one minute until the patient’s consciousness and eyelash reflex disappeared before proceeding with the operation. This protocol maximized the inhibition of stress and was the best time for inserting the fiberoptic bronchoscope. Therefore, the analgesic and sedative effects achieved in this study met the expectations of fiberoptic bronchoscopy doctors.

This study had several limitations. First, this was a small sample, single center clinical study, thus, further confirmation of the results is needed in a larger sample. Second, we only included PTB patients with ASA level II and excluded patients with other ASA levels. Finally, we did not rule out the impact of the severity of PTB on the study results. Despite these limitations, this study provided a reference for the ED50 of ciprofol in special populations of different ages and genders.

## Conclusion

This study showed that the ED50 of ciprofol combined with 0.15 μg/kg sufentanil for fiberoptic bronchoscopy in the four groups with PTB was 0.465 mg/kg, 0.433 mg/kg, 0.420 mg/kg and 0.396 mg/kg, respectively. The ED50 of ciprofol was significantly different among PTB patients of different genders and ages, which was lower in older patients than in non-elderly patients, and was lower in female patients than in male patients.

## Data Availability

The raw data of this study are available from the corresponding author on reasonable request.

## Appendix

### The formula for ED50

The numbers of satisfactory (r) and unsatisfactory cases (s) of fiberoptic bronchoscopy in pulmonary tuberculosis patients with different doses of ciprofol were determined. The logarithm (x) of each dose, the total number of patients (n), the satisfaction rate of bronchoscopy insertion (p), and the difference (I) between the logarithms of the two adjacent doses were calculated. The ED50 and its 95% CI were calculated using the following formula for half-dose sequential calculation:

$$P=r/\left(r+s\right)$$

Pair value lgED50 of ED50 = σ NX/σ n. Standard error of lgED50 (SlgED50) =$\text{I}\surd$ σ [p (1–p)/(n–1)] with 95% CI of ED50 pairs of values (lED50–1.96 SlgED50; lgED50 + 1.96 SlgED50.

## Abbreviations

ED50: Median effective dose
ED95: 95% Effective dose
PTB: Pulmonary tuberculosis
CSA: Chinese Society of Anesthesiology
ECG: Electrocardiogram
RR: Respiratory rate
BP: Blood pressure
SpO_2_: Oxygen saturation
ASA: American Society of Anesthesiologist
BMI: Body Mass Index
SBP: Systolic blood pressure
DBP: Diastolic blood pressure
HR: Heart rate

## References

[1] Khan FY, Aladab AH (2020). Role of fiberoptic bronchoscopy in the rapid diagnosis of sputum smear-negative disseminated tuberculosis with pulmonary miliary infiltrates. Oman Med J.

[2] Matot I, Kuras Y, Kramer MR (2000). Effect of clonidine premedication on haemodynamic responses to fibreoptic bronchoscopy. Anaesthesia.

[3] Brajer-Luftmann B, Mardas M, Stelmach-Mardas M, Lojko D, Batura-Gabryel H, Piorunek T (2021). Association between anxiety, depressive symptoms, and quality of life in patients undergoing diagnostic flexible video bronchoscopy. Int J Environ Res Public Health.

[4] Sharma N, Ahmed S (2022). Structural and functional pulmonary impairment in treated cases of pulmonary tuberculosis: a cross-sectional study. Med J Armed Forces India.

[5] Niemi M, Backman JT, Fromm MF, Neuvonen PJ, Kivistö KT (2003). Pharmacokinetic interactions with rifampicin: clinical relevance. Clin Pharmacokinet.

[6] Vrga B (2016). The contribution of the fiberoptic bronchoscopy in diagnosing of smear negative pulmonary tuberculosis. Lijec Vjesn.

[7] Quaiser S, Agarwal A, Khan R, Haque SF (2012). Fiberoptic bronchoscopy, as a valuable diagnostic option in sputum negative pulmonary tuberculosis: a prospective study. Int J Appl Basic Med Res.

[8] Liao J, Li M, Huang C, Yu Y, Chen Y, Gan J, Xiao J, Xiang G, Ding X, Jiang R (2022). Pharmacodynamics and pharmacokinetics of HSK3486, a novel 2,6-disubstituted phenol derivative as a general anesthetic. Front Pharmacol.

[9] Long YQ, Feng CD, Ding YY, Feng XM, Liu H, Ji FH, Peng K (2022). Esketamine as an adjuvant to ciprofol or propofol sedation for same-day bidirectional endoscopy: protocol for a randomized, double-blind, controlled trial with factorial design. Front Pharmacol.

[10] Bian Y, Zhang H, Ma S, Jiao Y, Yan P, Liu X, Ma S, Xiong Y, Gu Z, Yu Z (2021). Mass balance, pharmacokinetics and pharmacodynamics of intravenous HSK3486, a novel anaesthetic, administered to healthy subjects. Br J Clin Pharmacol.

[11] Hu Y, Li X, Liu J, Chen H, Zheng W, Zhang H, Wu M, Li C, Zhu X, Lou J (2022). Safety, pharmacokinetics and pharmacodynamics of a novel γ-aminobutyric acid (GABA) receptor potentiator, HSK3486, in Chinese patients with hepatic impairment. Ann Med.

[12] Li J, Wang X, Liu J, Wang X, Li X, Wang Y, Ouyang W, Li J, Yao S, Zhu Z (2022). Comparison of ciprofol (HSK3486) versus propofol for the induction of deep sedation during gastroscopy and colonoscopy procedures: a multi-centre, non-inferiority, randomized, controlled phase 3 clinical trial. Basic Clin Pharmacol Toxicol.

[13] Luo Z, Tu H, Zhang X, Wang X, Ouyang W, Wei X, Zou X, Zhu Z, Li Y, Shangguan W (2022). Efficacy and safety of HSK3486 for anesthesia/sedation in patients undergoing fiberoptic bronchoscopy: a multicenter, double-blind, propofol-controlled, randomized, phase 3 study. CNS Drugs.

[14] Wu B, Zhu W, Wang Q, Ren C, Wang L, Xie G (2022). Efficacy and safety of ciprofol-remifentanil versus propofol-remifentanil during fiberoptic bronchoscopy: a prospective, randomized, double-blind, non-inferiority trial. Front Pharmacol.

[15] Li X, Yang D, Li Q, Wang H, Wang M, Yan P, Wu N, Li F, Ma S, Ding Y (2021). Safety, pharmacokinetics, and pharmacodynamics of a single bolus of the gamma-aminobutyric Acid (GABA) receptor potentiator HSK3486 in healthy Chinese elderly and non-elderly. Front Pharmacol.

[16] Duan G, Lan H, Shan W, Wu Y, Xu Q, Dong X, Mei P, You M, Jin L, Wu J (2023). Clinical effect of different doses of ciprofol for induction of general anesthesia in elderly patients: a randomized, controlled trial. Pharmacol Res Perspect.

[17] Yang H, Zhao Q, Chen HY, Liu W, Ding T, Yang B, Song JC (2022). The median effective concentration of propofol with different doses of esketamine during gastrointestinal endoscopy in elderly patients: a randomized controlled trial. Br J Clin Pharmacol.

[18] Xu W, Drzymalski DM, Ai L, Yao H, Liu L, Xiao F (2021). The ED(50) and ED(95) of prophylactic norepinephrine for preventing post-spinal hypotension during cesarean delivery under combined spinal-epidural anesthesia: a prospective dose-finding study. Front Pharmacol.

[19] Liu H, Chen M, Lian C, Wu J, Shangguan W (2021). Effect of intravenous administration of lidocaine on the ED50 of propofol induction dose during gastroscopy in adult patients: a randomized, controlled study. J Clin Pharm Ther.

[20] Ludbrook G, Li F, Sleigh J, Liang Y. Assessments of onset and duration of drug effects and pharmacokinetics by dose level of HSK3486, a new sedative-hypnotic agent, in healthy female/male subjects: a phase I multiarm randomized controlled clinical trial. Anesth Analg. 2021;133(1):e16. 10.1213/ANE.0000000000005343 33464758

[21] Chen X, Guo P, Yang L, Liu Z, Yu D (2022). Comparison and clinical value of ciprofol and propofol in intraoperative adverse reactions, operation, resuscitation, and satisfaction of patients under painless gastroenteroscopy anesthesia. Contrast Media Mol Imaging.

[22] Lu M, Liu J, Wu X, Zhang Z (2023). Ciprofol: a novel alternative to propofol in clinical intravenous anesthesia?. Biomed Res Int.

[23] Zhu Q, Luo Z, Wang X, Wang D, Li J, Wei X, Tang J, Yao S, Ouyang W, Zhang W (2023). Efficacy and safety of ciprofol versus propofol for the induction of anesthesia in adult patients: a multicenter phase 2a clinical trial. Int J Clin Pharm.

[24] Qin X, Lu X, Tang L, Wang C, Xue J (2023). Ciprofol versus propofol for sedation in gastrointestinal endoscopy: protocol for a systematic review and meta-analysis. BMJ Open.

[25] Teng Y, Ou M, Wang X, Zhang W, Liu X, Liang Y, Li K, Wang Y, Ouyang W, Weng H (2021). Efficacy and safety of ciprofol for the sedation/anesthesia in patients undergoing colonoscopy: phase IIa and IIb multi-center clinical trials. Eur J Pharm Sci.

[26] Liu GL, Wu GZ, Ge D, Zhou HJ, Cui S, Gao K, Sun WJ, Yu DH, Liu SB, Liu JJ (2022). Efficacy and safety of ciprofol for agitation and delirium in the ICU: a multicenter, single-blind, 3-arm parallel randomized controlled trial study protocol. Front Med (Lausanne).

[27] Zhong J, Zhang J, Fan Y, Zhu M, Zhao X, Zuo Z, Zhou X, Miao C (2023). Efficacy and safety of Ciprofol for procedural sedation and anesthesia in non-operating room settings. J Clin Anesth.

[28] Qin K, Qin WY, Ming SP, Ma XF, Du XK (2022). Effect of ciprofol on induction and maintenance of general anesthesia in patients undergoing kidney transplantation. Eur Rev Med Pharmacol Sci.

[29] Ding YY, Long YQ, Yang HT, Zhuang K, Ji FH, Peng K (2022). Efficacy and safety of ciprofol for general anaesthesia induction in elderly patients undergoing major noncardiac surgery: a randomised controlled pilot trial. Eur J Anaesthesiol.

[30] Gembillo G, Cernaro V, Giuffrida AE, Russo G, Giandalia A, Siligato R, Longhitano E, Santoro D (2022). Gender differences in new hypoglycemic drug effects on renal outcomes: a systematic review. Expert Rev Clin Pharmacol.

[31] Man Y, Xiao H, Zhu T, Ji F (2023). Study on the effectiveness and safety of ciprofol in anesthesia in gynecological day surgery: a randomized double-blind controlled study. BMC Anesthesiol.

[32] Yang Y, Xia Z, Xu C, Zhai C, Yu X, Li S (2022). Ciprofol attenuates the isoproterenol-induced oxidative damage, inflammatory response and cardiomyocyte apoptosis. Front Pharmacol.

[33] Wang YC, Wu MJ, Zhou SL, Li ZH (2023). Protective effects of combined treatment with ciprofol and mild therapeutic hypothermia during cerebral ischemia-reperfusion injury. World J Clin Cases.

[34] Liu Y, Chen C, Liu N, Tong L, Nie Y, Wu J, Liu X, Gao W, Tang L, Guan X (2021). Efficacy and safety of Ciprofol sedation in ICU Patients with mechanical ventilation: a clinical trial study protocol. Adv Ther.

[35] Wang J, Li Y, Su H, Zhao J, Tu F (2022). Carotid artery corrected flow time and respiratory variations of peak blood flow velocity for prediction of hypotension after induction of general anesthesia in elderly patients. BMC Geriatr.

